# Interaction of carbohydrate-binding modules with poly(ethylene terephthalate)

**DOI:** 10.1007/s00253-019-09760-9

**Published:** 2019-04-16

**Authors:** Joanna Weber, Dušan Petrović, Birgit Strodel, Sander H. J. Smits, Stephan Kolkenbrock, Christian Leggewie, Karl-Erich Jaeger

**Affiliations:** 1 evoxx technologies GmbH, Alfred-Nobel-Str. 10, D-40789 Monheim am Rhein, Germany; 2 Institute of Molecular Enzyme Technology, Heinrich Heine University Düsseldorf, Forschungszentrum Jülich, D-52425 Jülich, Germany; 3 Present Address: Bayer AG, Friedrich-Ebert-Straße 475, 42117 Wuppertal, Germany; 4 Institute of Complex Systems ICS-6: Structural Biochemistry, Forschungszentrum Jülich GmbH, D-52425 Jülich, Germany; 5 Institute of Theoretical and Computational Chemistry, Heinrich Heine University Düsseldorf, Universitätstraße 1, D-40225 Düsseldorf, Germany; 6 Institute of Biochemistry, Heinrich Heine University Düsseldorf, Universitätsstraße 1, D-40225 Düsseldorf, Germany; 7 Present Address: Altona Diagnostics GmbH, Mörkenstr. 12, 22767 Hamburg, Germany; 8 Present Address: Erber Enzymes GmbH, Otto-Hahn-Straße 15, 44227 Dortmund, Germany; 9 Institute of Molecular Enzyme Technology, Institute of Bio- and Geosciences IBG-1: Biotechnology, Forschungszentrum Jülich GmbH, D-52425 Jülich, Germany

**Keywords:** Carbohydrate-binding modules, Poly(ethylene terephthalate) (PET), Functionalization, Surface affinity assay, Tryptophan quenching, Molecular dynamics

## Abstract

Poly(ethylene terephthalate) (PET) is one of the most widely applied synthetic polymers, but its hydrophobicity is challenging for many industrial applications. Biotechnological modification of PET surface can be achieved by PET hydrolyzing cutinases. In order to increase the adsorption towards their unnatural substrate, the enzymes are fused to carbohydrate-binding modules (CBMs) leading to enhanced activity. In this study, we identified novel PET binding CBMs and characterized the CBM-PET interplay. We developed a semi-quantitative method to detect CBMs bound to PET films. Screening of eight CBMs from diverse families for PET binding revealed one CBM that possesses a high affinity towards PET. Molecular dynamics (MD) simulations of the CBM–PET interface revealed tryptophan residues forming an aromatic triad on the peptide surface. Their interaction with phenyl rings of PET is stabilized by additional hydrogen bonds formed between amino acids close to the aromatic triad. Furthermore, the ratio of hydrophobic to polar contacts at the interface was identified as an important feature determining the strength of PET binding of CBMs. The interaction of CBM tryptophan residues with PET was confirmed experimentally by tryptophan quenching measurements after addition of PET nanoparticles to CBM. Our findings are useful for engineering PET hydrolyzing enzymes and may also find applications in functionalization of PET.

**Electronic supplementary material:**

The online version of this article (10.1007/s00253-019-09760-9) contains supplementary material, which is available to authorized users.

## Introduction

In the last few decades, plastic has taken a central role in the modern consumer society. With annually increasing production rates, poly(ethylene terephthalate) (PET) is one of the most frequently used synthetic polymers worldwide, with major applications as material for plastic bottles as well as in the textile and packaging industry (Zimmermann and Billig 2011; Köpnick et al. 2012; Wei and Zimmermann 2017). Crucial attributes are its high chemical resistance, mechanical strength, and low production costs (Zimmermann and Billig 2011). However, the hydrophobicity of PET presents a challenging feature for many applications as surfaces show low wettability and are difficult to functionalize (Espino-Rammer et al. 2013; Pellis et al. 2016). Conventional techniques used to modify PET require the application of harsh chemicals like concentrated alkali or plasma treatments to introduce hydrophilic groups (Brueckner et al. 2008; Tkavc et al. 2013). However, these chemical and physical methods exhibit a number of disadvantages, as they are environmentally detrimental or lead to loss in weight and bulk properties of the polymer (Shukla et al. 1997; Brueckner et al. 2008; Pellis et al. 2016).

Consequently, enzymatic treatment of PET also gained importance during the last years (Guebitz and Cavaco-Paulo 2008; Pellis et al. 2016). However, the usually poor affinity of various enzymes towards PET substrates represents a major challenge of enzymatic PET modifications (Pellis et al. 2016). To overcome this problem, PET-hydrolases (EC 3.1.1.101) are being fused to binding modules and hydrophobins (Espino-Rammer et al. 2013; Ribitsch et al. 2013; Zhang et al. 2013; Ribitsch et al. 2015), thus anchoring the enzyme on PET surfaces. In the present study, we focus on carbohydrate-binding modules (CBMs), which naturally function as non-catalytic domains in carbohydrate-active enzymes promoting substrate recognition and binding resulting in enrichment of the enzyme on the substrate surface (Boraston et al. 2004; Shoseyov et al. 2006). As polysaccharide targets are diverse in type, backbone linkage, and modification, CBM binding is promiscuous (Charnock et al. 2002; Popper 2008). CBMs are classified into different families based on amino acid sequence similarity and are further distinguished by their structural and functional properties giving rise to three types named A, B, and C (Boraston et al. 2004). Type A CBMs are surface-binding and comprise members of CBM families 1, 2a, 3, 5, and 10 (Boraston et al. 2004). A characteristic feature is their high binding capacity to insoluble, highly crystalline cellulose, and chitin, which is promoted by their platform-like binding site consisting of aromatic amino acid residues (Boraston et al. 2004). Previous studies already postulated an important role of surface exposed aromatic amino acids during interaction of CBMs with their synthetic substrate PET (Zhang et al. 2013).

Here, we have investigated CBM–PET interactions using a combined approach of in vitro and in silico studies to analyze CBM binding to PET. We have developed a fast and reliable PET surface affinity assay allowing for a semi-quantitative analysis of protein–PET interactions, thus enabling to screen various CBMs for PET binding. CBMs of different families possessing a planar architecture of aromatic acid residues were selected. Subsequently, three candidates were subjected to molecular dynamics (MD) simulations trying to reveal their mode of action and to correlate the experimentally determined affinity with the calculated models. Several recent studies described MD methods to examine the interactions of CBM1 from *Trichoderma reesei* with cellulose, which is a glucose-based polysaccharide (Nimlos et al. 2007; Beckham et al. 2010; Nimlos et al. 2012; Shiiba et al. 2013; Khazanov et al. 2016). There are, however, no atomistic MD studies of CBM interactions with other polymers like the synthetic PET. Based on the simulations and tryptophan quenching experiments, we propose a model describing CBM–PET interactions, which suggests residues and interactions responsible for binding.

## Materials and methods

### Construction of plasmids encoding CBMs

*E. coli* codon-optimized DNA sequences encoding for peptides *Tr*CBM1, *Pp*CBM1, *Ba*CBM2, *Bs*CBM2, *Ba*CBM5, ucCBM10, and *Pa*CBM10 as well as for fluorescence marker Bs2 (evoglow®; evoxx technologies, Monheim am Rhein, Germany) and the affinity tag *Strep*-tag® II (WSHPQFEK) were synthesized by GenScript® (Piscataway, USA). The DNA sequence encoding for peptide *Bl*CBM5 was kindly provided by the lab of Prof. Moerschbacher, University of Münster, Germany (Fuenzalida et al. 2014). Each sequence, encoding for one of the CBM peptides C-terminally fused to Bs2 and *Strep*-tag® II, was inserted into pET-22b(+) vector (Novagen, Merck, Darmstadt, Germany) lacking the *pelB* leader sequence. Cloning was performed using standard techniques as described previously (Sambrook et al. 1989) and Gibson Assembly® method. DNA was sequenced as custom service (GATC Biotech AG, Konstanz, Germany). Plasmids were transformed by heat shock protocol into chemical competent *E. coli* XL-1 Blue cells (Stratagene, San Diego, USA) and *E. coli* BL21 (DE3) [pLysS] cells (Stratagene, San Diego, USA) (Hanahan 1983).

### Expression and purification

Recombinant proteins were expressed in *E. coli* BL21 (DE3) [pLysS] (Stratagene, San Diego, USA) using the autoinduction method (Studier 2005). Cells were disrupted by sonication using Sonoplus Sonication Homogenisator (Bandelin, Berlin, Germany) adjusted to 3 × 5 min, 30% power, cycle 5. Soluble fractions were isolated by centrifugation (39,000 ×*g*, 30 min, 4 °C), and CBM fusion proteins were purified by affinity tag and ion exchange chromatography. For purification using affinity tag, the soluble fraction was loaded on a *Strep*-Tactin Superflow Plus (1 ml) cartridge (Qiagen, Hilden, Germany) equlibrated in washing buffer (20-mM triethanolamine, 300-mM NaCl, pH 8), and elution was performed using elution buffer (50-mM citrate, 100-mM Na_2_HPO_4_, 300-mM NaCl, 25-mM desthiobiotin, pH 5.2). For *Ba*CBM2-Bs2-StrepII, a different washing buffer (50-mM NaH_2_PO_4_, 300-mM NaCl, pH 8.0) and elution buffer (50-mM NaH_2_PO_4_, 800-mM NaCl, 25-mM desthiobiotin, pH 4.0) were used. Finally, the column was rinsed with 0.1-M NaOH to achieve complete elution. Proteins *Tr*CBM1-*Bs*2-StrepII, *Pp*CBM1-*Bs*2-StrepII, *Ba*CBM5-*Bs*2-StrepII, ucCBM10-*Bs*2-StrepII, and *Pa*CBM10-*Bs*2-StrepII were pre-purified by ion exchange chromatography using ÄKTApurifier (GE Healthcare, Chalfont St Giles, UK) before purification with affinity tag. The protein solution was desalted using 5-ml HiTrap Desalting Columns (GE Healthcare) and loaded on a 1-ml HiTrap Capto Q Column (GE Heathcare), equilibrated in washing buffer (50-mM triethanolamine, pH 7.5). The CBM fusion protein was eluted with an increasing gradient of elution buffer (50-mM triethanolamine, 1-M NaCl, pH 7.5) and tracked by a UV detector. After purification, buffer exchange and sample concentration were performed for all CBM fusion proteins using Vivaspin® 6 columns (Sartorius, Göttingen, Germany). The proteins were stored in 20-mM Tris/HCl pH 7. The protein concentrations of soluble fraction and purified protein solution were determined according to Bradford (1976), and protein samples were subjected to sodium dodecyl sulfate polyacrylamide gel electrophoresis (SDS-PAGE) using 12.5% acrylamide gels (Laemmli 1970). Gels were either used for Western Blot analyses or stained with Coomassie Brilliant Blue (Brunelle and Green 2014). For the latter, gels were incubated in Coomassie staining solution (0.05% Coomassie Brilliant Blue R250, 25% (*v*/*v*) isopropyl, 10% (*v*/*v*) acetic acid) for 1 h at 50-°C shaking. After washing with H_2_O, gels were incubated in destaining solution (10% (*v*/*v*) acetic acid) for at least 1 h at 50-°C shaking. For Western Blot analyses, gels were blotted onto Roti®-polyvinylidene difluoride (PVDF) membrane (Carl Roth, Karlsruhe, Germany) by electrotransfer (Towbin et al. 1979). Then, the Western Blot protocol of IBA Lifesciences (Göttingen, Germany) for chromogenic detection with alkaline phosphatase was conducted.

### PET surface affinity assay

In order to detect proteins on PET surfaces, a modified PET surface affinity assay based on a previously described enzyme adsorption assay (Ribitsch et al. 2013) was established. A biaxial-orientated PET film (Goodfellow, Huntingdon, UK, cat.no. ES301250) was washed with 10% (*w*/*v*) SDS (10 min with shaking) and with deionized H_2_O (2 × 10 min with shaking). The purified protein solution was applied on the PET film and incubated for 20 min. The PET film was then washed 3 × 5 min with Tris-buffered saline (TBS)-Tween buffer (50-mM Tris/HCl pH 7.4, 140-mM NaCl, 0.1% Tween 20). *Strep*-Tactin® AP conjugate (IBA Lifesciences, Göttingen, Germany) was added according to the manufacturer’s instructions. The PET film was washed with TBS-T and TBS (50-mM Tris/HCl pH 7.4, 140-mM NaCl) 2 × 1 min each. In parallel, a methanol-activated Roti®-PVDF membrane (Carl Roth, Karlsruhe, Germany) was soaked with 20-ml reaction buffer (100-mM Tris/HCl pH 8.8, 100-mM NaCl, 5-mM MgCl_2_) supplemented with the chromogenic substrate components NBT (7.5% *w*/*v* nitrotetrazolium blue in 70% *v*/*v* dimethylformamide; 10 μl) and BCIP (5% *w*/*v* 5-bromo-4-chloro-3-indolyl-phosphate in dimethylformamide; 60 μl). The PVDF membrane was then placed on the PET film for the chromogenic reaction, which was stopped by washing with H_2_O. The dried PVDF membrane was then scanned, and spots of equal size were computationally cut out and analyzed by densitometry using the software ImageJ (Schindelin et al. 2015).

### Production of PET nanoparticles

PET nanoparticles were produced using a modified version of a previously described protocol (Welzel et al. 2002; Pütz 2006). One-hundred-milligram PET dissolved in 10-ml 1,1,1,3,3,3,-hexafluor-2-propanol was filled in a burette. Under vigorous stirring at 1000 rpm using an overhead stirrer (RZR 2051, Heidolph, Schwabach, Germany), the solution was dropped into a prechilled ice-cold three-neck round-bottom flask filled with 100-ml deionized H_2_O. The precipitated polymer was filtered using filter paper 305 (VWR, Rednor, USA). Organic solvent was evaporated for 3 days and removed by concentrating the suspension using a vacuum concentrator (Concentrator 5301, Eppendorf, Hamburg, Germany). The PET nanoparticle concentration was gravimetrically determined as mean value of dried PET mass obtained after evaporation of 5 × 1-ml PET nanoparticle suspension. The average size distribution of the nanoparticles was in the range between 50 and 100 nm as determined using a Zeta Sizer and by transmission electron microscopy.

### Tryptophan quenching

For the determination of tryptophan quenching, three different experimental runs (denoted by the letters a, b, c) were carried out. Each sample was excited at 280 nm after 30 s of incubation in a cuvette, and the emission spectrum was recorded from 300 to 400 nm using spectrofluorometric fluorolog® (Horiba Scientific, Kyoto, Japan) (Teale and Weber 1957; Ghisaidoobe and Chung 2014). (*a*) PET nanoparticles were added in 5-ng steps in a volume of 5 μl to a solution of 10-μg purified *Ba*CBM2-Bs2-StrepII in 1-ml 20-mM Tris/HCl pH 7. (*b*) PET nanoparticles were added in 5-ng steps in a volume of 5 μl to a solution of 1-ml 20-mM Tris/HCl pH 7. (*c*) Deionized H_2_O was added in 5-μl steps to a solution of 10-μg purified *Ba*CBM2-Bs2-StrepII in 1-ml 20-mM Tris/HCl pH 7 as a control to determine the dilution effect. The fluorescence counts at 354 nm from each measuring point of the experimental runs (*a*, *b*, *c*) were used for the calculations. The normalized change in fluorescence (*z*%) was determined using the following equations:

$$ (a)\hbox{--} (b)=x\kern0.5em \mathrm{subtraction}\ \mathrm{of}\ \mathrm{PET}\ \mathrm{nanoparticle}\ \mathrm{fluorescence} $$

$$ x\ast \mathrm{volume}=y\kern0.5em \mathrm{dilution}\ \mathrm{by}\ \mathrm{adding}\ \mathrm{PET}\ \mathrm{nanoparticles} $$

$$ y\%-(c)\%=z\%\kern0.5em \mathrm{subtraction}\ \mathrm{of}\ \mathrm{changes}\ \mathrm{in}\kern0.50em \mathrm{tryptophan}\ \mathrm{fluorescence}\ \mathrm{without}\ \mathrm{PET}\ \mathrm{nanoparticles} $$

### Alignments and homology modeling

Amino acid sequences of CBMs were aligned using Clustal Omega (Goujon et al. 2010; Sievers et al. 2011) and ESPript 3.0 (Robert and Gouet 2014). The initial peptide conformations were determined by homology modeling using SWISS-MODEL (Biasini et al. 2014). The most reliable homology model, based on the highest sequence identity and best Global Model Quality Estimation value, was selected for each candidate, and the structure was further evaluated by inspection of the Ramachandran Plot (Lovell et al. 2003) and VADAR analysis (Willard et al. 2003).

### MD simulations of CBMs in solution

Peptide topologies of the homology models were created with the CHARMM27 force field (MacKerell et al. 2004) and solvated with TIP3P water (Jorgensen et al. 1983) in a cubic box. Protonation states were determined based on the PROPKA 3 analysis (Olsson et al. 2011), and all titratable residues were assigned to a state corresponding to pH 7. Disulfide bonds were established when the sulfur atoms of two cysteines were located within the common cutoff of 2 Å. Sodium chloride was added yielding a 40-mM concentration. After 2000 steps of energy minimization, all systems were equilibrated in the NPT (isothermal-isobaral) ensemble with the Berendsen barostat (Berendsen et al. 1984) for 5 ns (2-fs time step), with restraints on the peptide atoms being scaled down from initially 5 to 0.5 kcal mol^−1^ Å^−2^ at the end of the simulation. Unrestrained production MD was run for 100 ns in the NVT (isothermal-isochoric) ensemble, and coordinates were saved every 10 ps. The Langevin thermostat was used to maintain a constant temperature of 300 K. The cutoff distance for the short-range non-bonded interactions was 12 Å, with a switching function turned on at 10.5 Å. Simulations were performed using ACEMD (Harvey et al. 2009), a highly optimized GPU molecular dynamics code. The same MD parameters (cutoffs, time step, periodic boundary conditions with the particle-mesh Ewald method for the calculation of electrostatic interactions, thermostat, and barostat) were used for all subsequent MD simulations involving PET.

### Constructing the PET surface for MD

The PET chains were built from the fiber structure determined in *P*-1 space group (Fu et al. 1993). Each PET chain consisted of five monomer units and eight PET chains formed a layer large enough to study peptide binding. Five such layers were then stacked on top of each other yielding the PET surface, which was aligned along the x- and y-axes. The PET topology was created with the CHARMM force field (Cruz-Chu et al. 2009) in a way that the last (fifth) residue in a chain is connected to the first one of that chain, enabling to simulate an infinitely long polymer. A water box of 90-Å height was added on top of the PET surface, leading to ~ 26,000 atoms in total. The system was energy-minimized for 2000 steps and further equilibrated using MD simulations in two NPT phases: (1) 5 ns with 5-kcal mol^−1^ Å^−2^ restraints on the PET atoms followed by (2) 10 ns without any restraints. The final frame of the equilibrated system was used for modeling peptide binding to PET (Online Resource Fig. S1a–c).

### MD simulations of CBM binding to PET

To simulate the CBM–PET interactions, we used the homology models of *Tr*CBM1, *Ba*CBM2, and *Ba*CBM5 and the corresponding topologies that were employed for the simulations in water. In addition, we performed the alanine scanning of the aromatic triad of *Ba*CBM2, simulating the W9A/W44A/W63A *Ba*CBM2 mutant. In each case, the peptide was centered over the PET surface and positioned ~ 10 Å away, with the coplanar aromatic triad oriented parallel to the surface (Online Resource Fig. S1d). These simulations were run in triplicate with different random positions for the three initial conformations. Water molecules located within 2.4 Å of any peptide atom were removed, and sodium chloride was added yielding a 40-mM concentration. The system was energy-minimized for 2000 steps, followed by a 2-ns equilibration in the NPT ensemble, where in the first 0.5 ns, all peptide atoms were restrained with 5-kcal mol^−1^ Å^−2^ force and the first 1 ns was performed with the time step of 1 fs to enable smooth equilibration. Each production MD was run in the NVT ensemble for 100 ns.

### Analysis of the MD simulations

VMD 1.9.1 (Humphrey et al. 1996), GROMACS 4.6.7 (Van Der Spoel et al. 2005), DSSP (Kabsch and Sander 1983), and Python 3 were used for the analysis of the MD trajectories. Graphical rendering was performed with VMD and PyMOL (The PyMOL Molecular Graphics System, Version 1.6 Schrödinger, LCC).

### Accession numbers and PDB codes

Codon-optimized DNA sequences of CBMs used in this study are deposited with the following GenBank accession numbers (# of amino acid sequence of originating protein/# of DNA-sequence): ADC83999.1/MK349004 (*Tr*CBM1), CAY71902.1/MK349003 (*Pp*CBM1), ACQ50287/MK349005 (*Ba*CBM2), BAM53958/MK349006 (*Bs*CBM2), AAU21943.2/MK349008 (*Bl*CBM5), ABI86195.1/MK349007 (*Ba*CBM5), AAP49340.1/MK349010 (ucCBM10), and ACX31080.1/MK349009 (*Pa*CBM10). The protein sequence of the fluorescence marker Bs2 has the following GenBank accession number: ABN71355.1. The PDB codes of the templates used to generate the initial peptide conformations with homology modeling are 4bmf.1.A (*Tr*CBM1), 4qi5.1.A (*Pp*CBM1), 3ndy.1.B (*Ba*CBM2), 2rtt.1.A (*Bs*CBM2), 4hme.1.A (*Ba*CBM5), 4hme.1.A (*Bl*CBM5), 1e8r.1.A (*Pa*CBM10), and 1e8r.1.A (ucCBM10).

## Results

### Identification of CBMs for PET binding

**Fig. 1:**
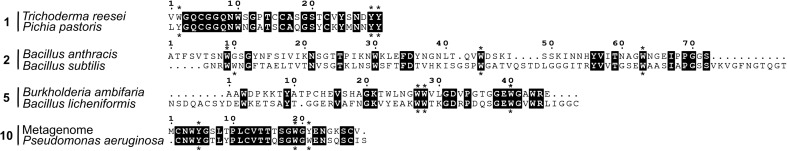
Amino acid sequence alignments and origin of selected CBMs. Identical residues are highlighted by a black background. The amino acids of each predicted aromatic triad are labeled with asterisks

Two members per family 1, 2, 5, and 10 belonging to type A CBMs were chosen from the Carbohydrate Active Enzymes database (http://www.cazy.org/) (Cantarel et al. 2009; Lombard et al. 2014). Sequence alignments revealed moderate to high sequence similarity within the families, while sequences from different families varied substantially (Fig. 1).

**Fig. 2:**
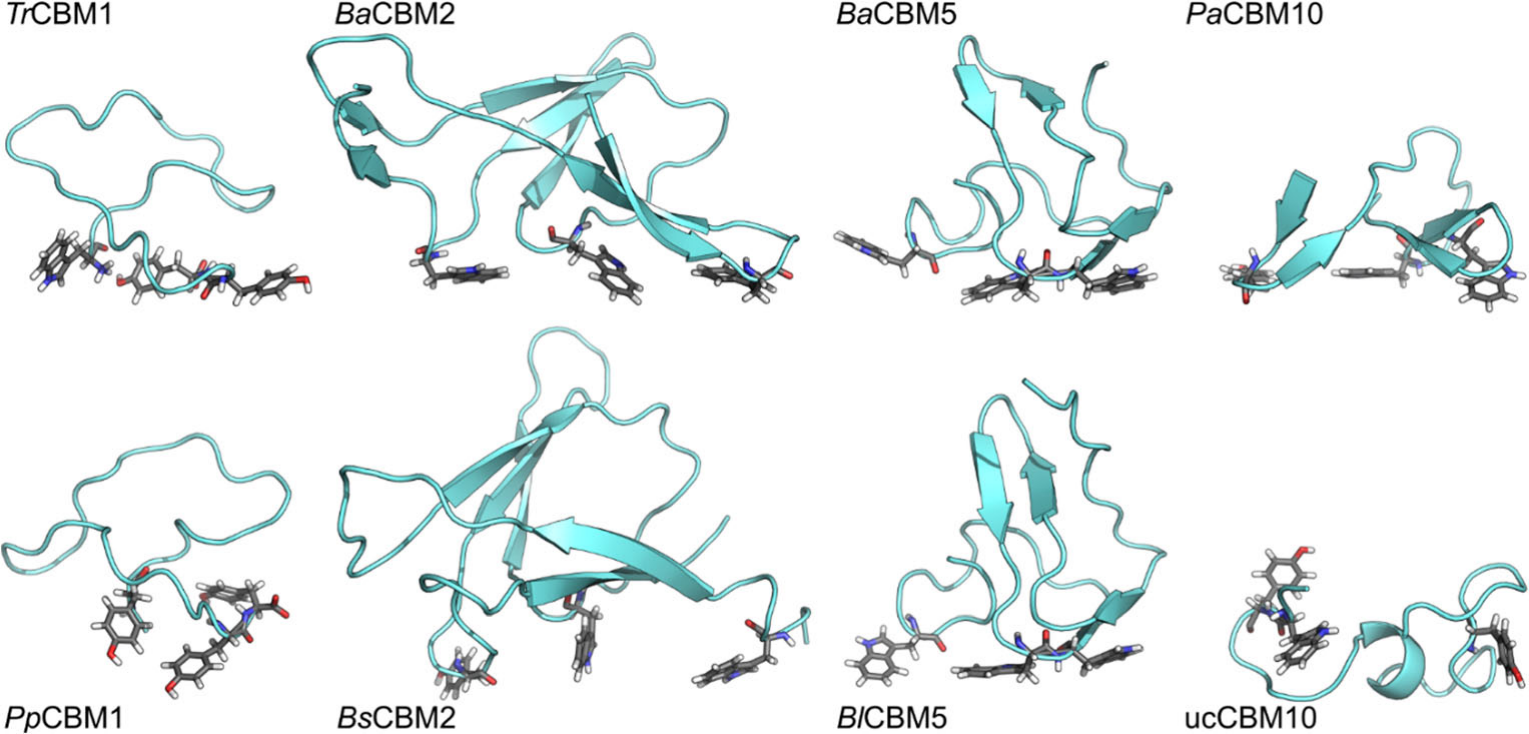
Representative structures of eight selected peptides obtained from MD simulations in bulk water. The orientation of the aromatic residues forming a triad, as anticipated from homology modeling, are shown as sticks. The simulation of *Bs*CBM2 was performed using a variant W63Y

The initial peptide conformations were determined by homology modeling. Peptide MD simulations in bulk water were performed to confirm the quality and stability of the proposed homology models (Fig. 2). The selected peptides can be divided into two groups based on their size: (1) CBM2 and CBM5 peptides possess more than 40 residues and (2) CBM1 and CBM10 peptides comprise less than 30 amino acids. The group 1 peptides showed a low root-mean-square deviation (RMSD) of the backbone atoms, typically in the range of 1–3 Å. The RMSD indicates how much a peptide deviates from its initial structure during the course of an MD simulation. A low RMSD is typically an indicator of a stable peptide structure. Another stability parameter is the radius of gyration (Rg) that correlates with the overall shape of a peptide. The group 1 peptides are characterized by stable Rg values over the entire simulation—a characteristic of a folded protein. Group 2 peptides behaved significantly different, showing a high level of motion indicated by the elevated RMSD values, typically > 4 Å. They are also characterized by fluctuating Rg values, which implies the existence of local folding and unfolding events. For instance, ucCBM10 folded to a helix during the MD simulation, which subsequently unfolded again (Fig. 2). The folding processes were confirmed by the analysis of the secondary structure development over the simulation trajectories.

### Cloning, expression, and purification of recombinant proteins

Synthetic genes of selected CBMs were codon-optimized for *E. coli* and cloned into pET 22b(+) vector. For rapid detection and purification, each CBM was C-terminally fused to the fluorescence marker Bs2 and the affinity tag *Strep*-tag® II. Auto-induced expression of the peptides was carried out in *E. coli* BL21 (DE3) [pLysS]. SDS-PAGE and subsequent Western Blot analysis (data not shown) indicated that transformants produced soluble recombinant proteins of molecular mass corresponding to the calculated values. Recombinant proteins *Ba*CBM2-Bs2-StrepII and *Bl*CBM5-Bs2-StrepII were purified by *Strep*-tag® II technology to high purity (Online Resource Fig. S2) with yields in the range of 30–800 μg/L. The remaining recombinant proteins were purified by additional ion exchange chromatography still varying in purity (Online Resource Fig. S2).

### PET surface affinity assay

**Fig. 3:**
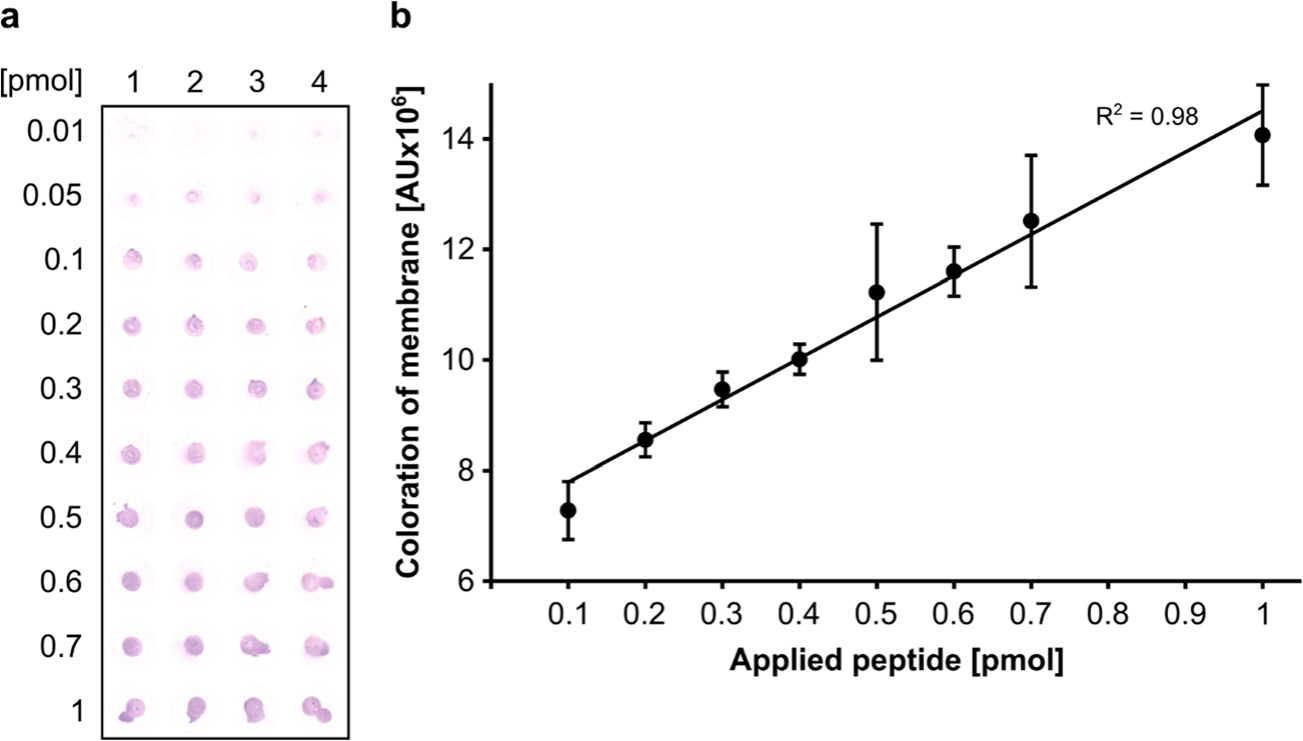
PET surface affinity assay. **a** Replica-membrane of PET surface treated with increasing amounts of the purified peptide *Ba*CBM2-Bs2-StrepII in four replicas. Each spot of the scanned membrane was cut out computationally in order to avoid background signals in densitometric analysis. **b** Densitometric analysis of membrane spots from **a** using ImageJ (26)

The developed PET surface affinity assay is based on the detection of recombinant protein on PET film via its affinity tag *Strep*-tag® II. Alkaline phosphatase labeled with *Strep*-Tactin® binds *Strep*-tag® II C-terminally fused to CBM, which in turn is immobilized on the PET film. The alkaline phosphatase hydrolyzes its chromogenic substrate located into a PVDF membrane, which is positioned onto the PET film. The resulting colored product is retained on the PVDF membrane (output signal). Densitometric analysis of the scanned PVDF membrane showed a linear correlation between the amount of PET binding protein *Ba*CBM2-Bs2-StrepII and the intensity of the output signal in a range of 0.1 to 1 pmol (Fig. 3a, b). Application of more than 1 pmol did not result in a further linear increase of the coloration (data not shown) indicating saturation of density or PET coating.

### CBM screening for PET binding

**Fig. 4:**
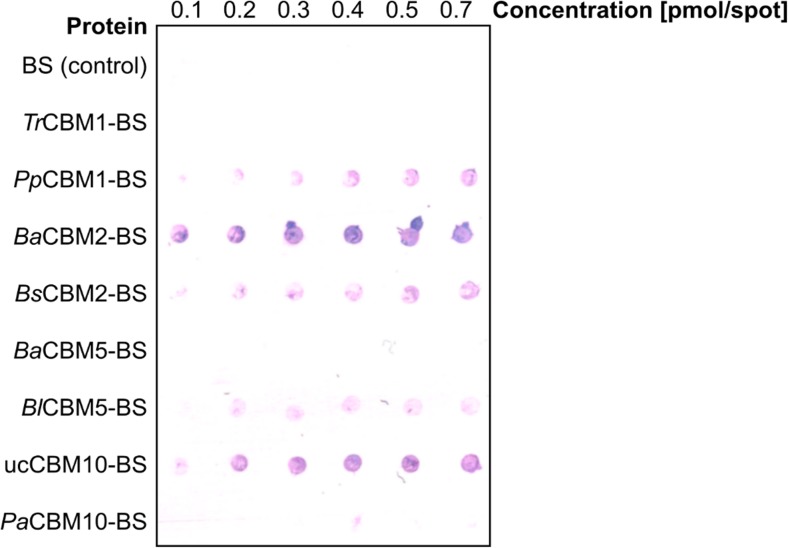
Screening of different CBMs for PET binding. Concentrated protein solutions of CBM fusion proteins were applied on PET surface (BS: Bs2-StrepII Tag). The replica-membrane of the PET surface is pictured, and the colored spots indicate CBMs bound to PET

CBMs were screened for PET binding using the PET surface affinity assay by applying each CBM fusion protein and the negative control Bs2-StrepII without CBM at different concentrations on a PET film (Fig. 4). The output signals indicated that at least 5 out of 8 CBMs bind the synthetic substrate PET. Moreover, the intensities of the output signals varied indicating differences in PET binding affinities. It should be noted that the amount of applied CBMs could not be exactly quantified due to varying degrees of protein purity of the samples (Online Resource Fig. S2). Nevertheless, the PET surface affinity assay allowed to identify CBMs binding to PET. Furthermore, *Ba*CBM2 showed the strongest output signal indicating superior binding affinity. Apparently, *Tr*CBM1, *Ba*CBM5, and *Pa*CBM10 did not bind to PET. Here, the amount of bound protein may be below the detection limit or these CBMs do not bind to PET at all.

### Insights into the interactions at the CBM–PET interface from MD simulations

**Fig. 5:**
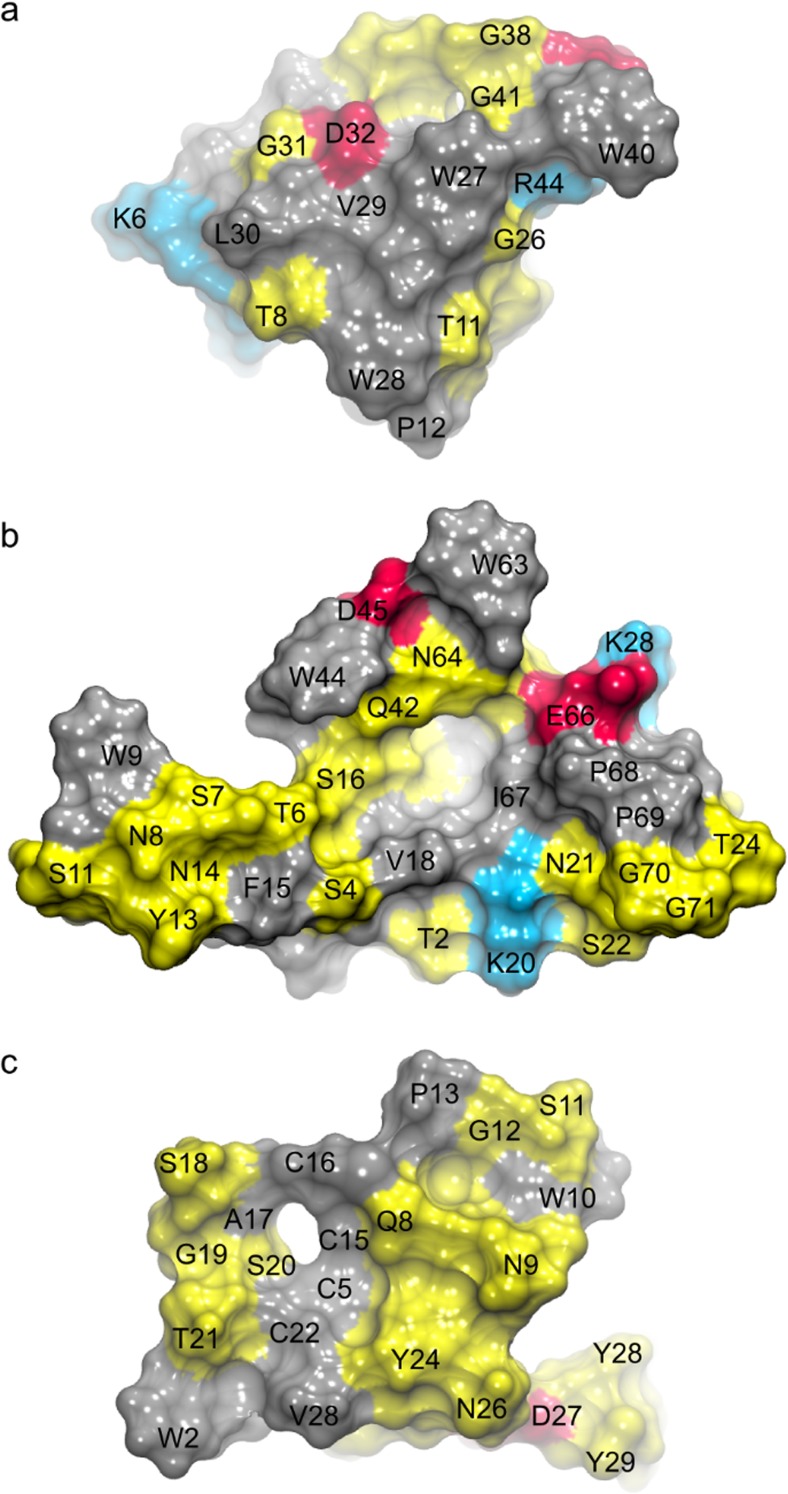
The computationally predicted constellation of residues in **a***Ba*CBM5, **b***Ba*CBM2, and **c***Tr*CBM1 that are in direct contact with PET (view of the protein from the PET interface). Hydrophobic residues are shown in gray, polar in yellow, acidic in red, and alkaline in blue. The contacts from the three MD runs per peptide are shown in Online Resource Fig. S5

**Fig. 6:**
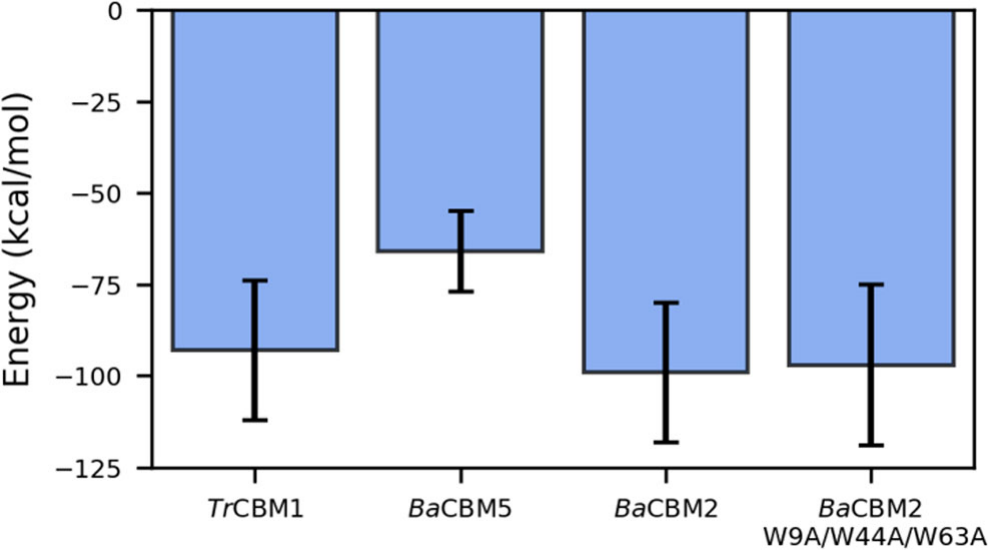
The average CBM-PET potential energy from the last 10 ns of the triplicate simulations. The energy is the sum of the van der Waals and electrostatic contributions. For the energy profiles over complete simulations, see Online Resource Fig. S4

For the further computational investigation of the CBM–PET interactions, we selected representatives from three different CBM families, namely *Tr*CBM1, *Ba*CBM2, and *Ba*CBM5 (Fig. 5). The residues Trp27/Trp28/Trp40 of *Ba*CBM5 formed an aromatic triad mediating π-stacking interactions with the phenyl rings of PET. The peptide structure was rigid (RMSD < 2 Å, Online Resource Fig. S3) and, due to intrinsic geometrical properties, only a small number of residues were interacting with the PET surface (Fig. 5a). The interaction energy between the peptide and PET dropped quickly as the aromatic triad approached the PET surface (Online Resource Fig. S4). Although several polar or charged residues were in the vicinity of the interface, none of them could find an optimal arrangement of the side chains to form stabilizing H-bonds with the surface. Therefore, the energy remained quite high compared with other systems (Fig. 6). It should be noted that the smaller (more negative) the energy is, the stronger is the binding. The MD result agrees with the finding from the in vitro assay, which could not detect PET binding of *Ba*CBM5.

*Ba*CBM2, although only slightly larger than *Ba*CBM5, has a very different amino acid composition that drastically changes the response to PET exposure. The aromatic triad consists of Trp9/Trp44/Trp63. Here, Trp9, Trp44, and the surrounding polar amino acids readily approached the PET surface (Fig. 5b) to establish both π-stacking interactions and H-bonds. The initial binding to PET induced a conformational change in *Ba*CBM2, which further enabled a simultaneous access of Trp63 and Asn64 to the surface. The second binding step (at ~ 25 ns) significantly decreased the interaction energy (Fig. 6). The aromatic triad predicted from the homology model of *Tr*CBM1 (i.e., Trp2/Tyr28/Tyr29) is not involved in π-stacking interactions with PET. In our simulations, although initially close to the surface, Tyr28 and Tyr29 quickly detached from it. After a structural rearrangement of *Tr*CBM1, a new aromatic triad consisting of Trp2/Trp10/Tyr24 or Trp2/Tyr24/Tyr29 (Online Resource Fig. S5) formed. However, Trp10 was beyond the common cut-off to form a strong π-interaction with PET (Fig. 5c). Unlike the very hydrophobic contact surface of *Ba*CBM5 or the nicely blended hydrophobic and polar surface of *Ba*CBM2, the contact area of *Tr*CBM1 was highly polar, leading to a similar binding strength between *Tr*CBM1 and PET in the MD simulations as observed for *Ba*CBM2 (Fig. 6). However, *Tr*CBM1 is very flexible. In bulk water, it lacked a well-defined shape and secondary structures (Online Resource Figs. S6 and S7). Apart from a transient helix that existed for 10 ns in the MD simulation in water, there were no stabilizing intramolecular interactions, and the structure was completely coiled. Upon binding to the PET surface a conformational change in *Tr*CBM1 can be induced, leading to the formation of a β-sheet as a consequence of the π-stacking interactions of Trp2 and Tyr24 with the PET surface (Online Resource Figs. S7 and S8). The conformational flexibility of *Tr*CBM1 may counteract its binding to PET on a longer timescale as no binding was observed in the PET surface affinity assay under current experimental conditions. Moreover, compared with the binding of *Ba*CBM2, which in each of the three MD runs largely bound with the same amino acid residues to PET, the flexibility of *Tr*CBM1 leads to a less specific binding as this peptide invoked different residues when binding to the PET surface in the three MD runs (Online Resource Fig. S5).

### Tryptophan residues of CBMs are important for PET binding

**Fig. 7:**
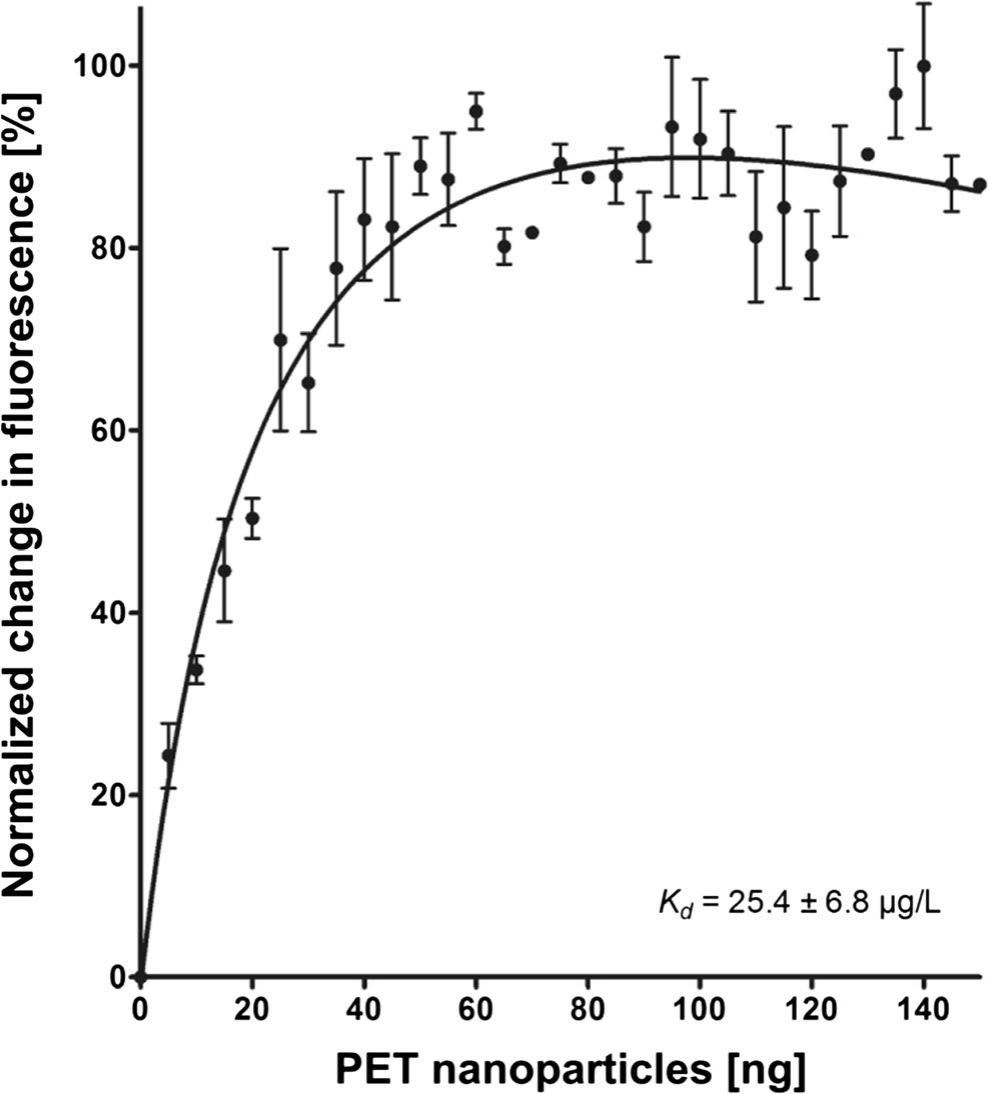
Tryptophan quenching of *Ba*CBM2-Bs2-StrepII by PET nanoparticles. The estimated *K*_d_ is 25.4 ± 6.8 μg/L.

In order to verify the hypothesis that tryptophan residues play an important role in PET interaction, we carried out tryptophan quenching experiments using the PET binding fusion protein *Ba*CBM2-Bs2-StrepII and PET nanoparticles. The data show that increasing amounts of PET nanoparticles resulted in increased quenching of normalized tryptophan fluorescence (Fig. 7), until saturation was reached at a concentration of ≥ 60 ng. This result indicates that tryptophan residues interact with PET nanoparticles, which is in agreement with our predictions from the MD simulations showing that Trp9, Trp44, and Trp63 bind to the PET surface. If these three residues are mutated to alanine, the PET binding at position 44 is completely lost, while Ala63 binds less than Trp63 (Online Resource Fig. S5). The tryptophan fluorescence data allowed to calculate an apparent dissociation constant *K*_d_ of 25.4 μg/L.

## Discussion

PET binding peptides can serve to improve the interaction of PET hydrolyzing enzymes with their substrate (Ribitsch et al. 2013; Zhang et al. 2013). Moreover, they could be applied as adaptors on synthetic polymer surfaces, offering various innovative application possibilities for the coating of packaging to increase the shelf-life of edibles, the immobilization of water-soluble proteins on polymer surfaces, or the functionalization of synthetic fibers, e.g., by conjugation of pigments (Guebitz and Cavaco-Paulo 2008; Noor et al. 2012; Karam et al. 2013). Thus, the identification of PET binding peptides as well as a better understanding of the binding mode represent current challenges in protein-polymer research. Previous studies had indicated that CBMs are potential candidates for PET binding peptides, since their fusion to PET hydrolases improved the activity of these enzymes, most likely by strengthening their affinity to the substrate (Ribitsch et al. 2013; Zhang et al. 2013). CBMs are well studied binding modules, which can be easily produced, in contrast to, for example, antimicrobial peptides, which are also candidates for synthetic polymer binding (Noor et al. 2012; Rübsam et al. 2017), but their recombinant production is difficult (Parachin et al. 2012).

Eight CBMs belonging to different CBM families were screened for PET binding. These CBMs were chosen according to the presence of a planar architecture of surface exposed aromatic residues. Peptide structures were predicted by homology modeling, and the model quality was evaluated by MD simulations. The proposed structures for CBM2 and CBM5 peptides were stable in water, and their characteristics are comparable to those of other peptides of this size (50–100 residues). The peptides from families CBM1 and CBM10 are, however, very flexible and susceptible to drastic changes in both secondary and tertiary structure, suggesting that a major and stable fold does not exist. As the latter peptides are very short (approx. 30 residues), it is not surprising that they are unstructured and flexible in bulk water as opposed to the stable fold that the homologous CBMs generally adopt when they are part of larger enzyme structures, where surrounding domains can stabilize the specific CBM fold.

PET binding affinity of the selected CBMs was analyzed in vitro after fusion to fluorescence markers to increase the solubility of the fusion protein and to track CBMs during purification. Furthermore, the fluorescence marker served as a linker between the *Strep*-tag® II and the CBM to ensure equal accessibility of the alkaline phosphatase to the *Strep*-tag® II. An assay to study enzyme adsorption based on HisProbe-HRP (Thermo Scientific, Waltham, USA) detection has been used in a previous study to determine the binding affinity of the cutinase Thc_Cut1 and its fusion to a CBM and a polyhydroxyalkanoate binding module (Ribitsch et al. 2013). Here, a modified protocol of this assay was established, which enables the semi-quantitative determination of PET affinities as the applied protein amount is proportional to the intensity of the output signal, though only output signals on the same membrane can be compared with each other. The purification of the CBM fusion proteins turned out to be highly challenging, as the tested constructs bound to various polymers used in purification columns. Due to differences in final purity of the fusion proteins as indicated by a varying number of additional protein bands revealed by SDS-PAGE analysis (Online Resource Fig. S2), a quantitative determination of the affinity of the CBM fusion proteins to PET was not possible. Nonetheless, this assay provided a qualitative and reliable measure of PET binding properties and thus allowed the identification of PET binding CBMs. *Ba*CBM2 attracted our attention as its expression rate was relatively high, and the fusion protein could be prepared with high purity. Moreover, in contrast to *Bl*CBM5 with comparable purity, *Ba*CBM2 turned out to possess the highest affinity to PET among the selected peptides indicating its potential for industrial applications.

MD simulations of PET binders and non-binders based on the results from the PET surface affinity assay were carried out to identify the key residues and their local orientation responsible for binding. The investigated peptides *Ba*CBM2 and *Ba*CBM5 contain a surface-exposed aromatic triad, which participates in the interaction with PET. The simulations revealed that this interaction is driven by both π-stacking interactions with the phenyl rings of PET and polar interactions, especially hydrogen bonds that formed between the CBMs and the PET ester groups.

The interactions of CBMs with their synthetic substrate PET were experimentally analyzed too. Tryptophan quenching is a convenient biochemical parameter for determining protein–ligand interaction as the tryptophan fluorescence maximum and intensity are highly influenced by changes in its micro-environment (Ghisaidoobe and Chung 2014). Hence, we conducted tryptophan quenching experiments revealing that *Ba*CBM2 fusion protein interacts with PET as tryptophan fluorescence changed by addition of PET nanoparticles. Tryptophan quenching also indicates that tryptophan residues are themselves involved in this interaction; however, it remains elusive which tryptophan residues contribute to binding. Apart from the tryptophans of *Ba*CBM2, the fusion protein and the spacer carry three more tryptophans. As the control protein Bs2-StrepII, which possesses two tryptophan residues did not show any PET affinity, it can be assumed that tryptophans involved in quenching belong to *Ba*CBM2. This method enabled the first reported determination of a dissociation constant (*K*_d_ = 25.4 μg/L) for CBM–PET interaction, thus offering a means to quantitatively compare affinities of different CBMs to PET. It should be noted that the tryptophan quenching experiments required to use PET nanoparticles of a mainly amorphous structure, which is different from the ordered PET surfaces used for MD simulations and in the PET surface affinity assays.

The MD simulations revealed that the ratio between hydrophobic and polar interactions at the CBM–PET interface seems to be an important feature to distinguish between high and low affinity to PET. The peptides *Ba*CBM5 and *Tr*CBM1 did not show binding to PET using the surface affinity assay. Although we are aware of the fact that less protein was applied to the PET film as compared to *Ba*CBM2, in case of *Ba*CBM5, the MD simulation revealed a mainly hydrophobic contact surface. We hypothesize that this interaction is not strong enough for permanent binding, supported by the relatively high energy, so that *Ba*CBM5 is probably washed away during the washing procedure of the PET surface affinity assay. In case of *Tr*CBM1, the MD simulation revealed a small and mainly polar contact area, which facilitates binding to the PET surface. However, *Tr*CBM1 is not structurally stable and undergoes large conformational transitions both in bulk water and when bound to PET. This conformational flexibility probably counteracts stable binding of this CBM peptide to PET on a larger scale as seen in the PET surface affinity assay. In contrast to the extremes *Tr*CBM1 and *Ba*CBM5, an excellent balance between hydrophobic and polar interactions, in addition to a greater contact area, was found for *Ba*CBM2 leading to a low energy in silico (Fig. 6). This finding is in perfect agreement with the PET binding observed in the in vitro assay (Fig. 4). The importance of hydrogen bonds, in addition to π-interactions, between tryptophans and ester bonds in PET was previously reported (Zhang et al. 2013). Zhang et al. hypothesized that the exchange of W68Y in CBM_CenA_ fused to a cutinase leading to an enhanced binding capacity was due to formation of a new hydrogen bond between the hydroxyl group of tyrosine and PET.

In conclusion, we have developed a fast and reliable screening method to identify experimentally PET binding peptides. Furthermore, MD simulations of the CBM–PET interface revealed π-stacking interactions of exposed aromatic residues of the peptide with the phenyl rings of PET and hydrogen bonding of polar residues. We conclude that a well-balanced contact area of hydrophobic and polar residues positively affects the strength of CBM–PET binding. These novel findings can be applied to engineer CBM sequences for improved PET binding and to evaluate possible binding modules in silico. Additionally, MD simulation turned out to be a convenient method for the elucidation of the PET binding mechanism and is thus a promising method for a streamlined optimization of CBM binding properties. Thus, next steps could be the integration of tryptophans and hydrogen bond forming amino acids in *Ba*CBM2 and its biotechnological application as fusion partner to PET hydrolyzing enzymes.

## Electronic supplementary material

ESM 1 (PDF 1291 kb)
