# Supplementary material for: Interaction of carbohydrate-binding modules with poly(ethylene terephthalate)
Source: Appl Microbiol Biotechnol. 2019 Apr 16;103(12):4801–12. doi: 10.1007/s00253-019-09760-9 (PMC6536475; doi:10.1007/s00253-019-09760-9)
Supplement: Supplementary file 1 — (PDF 1291 kb) [file 253_2019_9760_MOESM1_ESM.pdf]

# Supporting Information

**Journal:** Applied Microbiology and Biotechnology

**Title:** Interaction of carbohydrate-binding modules with poly(ethylene terephthalate)

**Authors:** Joanna Weber[a,b1], Dušan Petrović[c], Birgit Strodel[c,d], Sander H.J. Smits[e], Stephan Kolkenbrock[a2], Christian Leggewie[a3*], Karl-Erich Jaeger[b,f,*]

[a] evoxx technologies GmbH, Alfred-Nobel-Str. 10, D-40789 Monheim am Rhein, Germany

[b] Institute of Molecular Enzyme Technology, Heinrich Heine University Düsseldorf, Forschungszentrum Jülich, D-52425 Jülich, Germany

[c] Institute of Complex Systems ICS-6: Structural Biochemistry, Forschungszentrum Jülich GmbH, D-52425 Jülich, Germany

[d] Institute of Theoretical and Computational Chemistry, Heinrich Heine University Düsseldorf, Universitätstraße 1, D-40225 Düsseldorf, Germany

[e] Institute of Biochemistry, Heinrich Heine University Düsseldorf, Universitätsstraße 1, D-40225 Düsseldorf, Germany

[f] Institute of Bio- and Geosciences IBG-1: Biotechnology, Forschungszentrum Jülich GmbH, D-52425 Jülich, Germany

[1] Present address: Bayer AG, Friedrich-Ebert-Straße 475, 42117 Wuppertal, Germany
[2] Present adress: Altona Diagnostics GmbH, Mörkenstr. 12, 22767 Hamburg, Germany
[3] Present address: Erber Enzymes GmbH, Otto-Hahn-Straße 15, 44227 Dortmund, Germany

***Corresponding authors:**

Dr. Christian Leggewie

Erber Enzymes GmbH

Otto-Hahn-Str. 15

D-44227 Dortmund, Germany

Phone: +49 231 97979408

Email: christian.leggewie@erber-group.net

Prof. Dr. Karl-Erich Jaeger

Institut für Molekulare Enzymtechnologie

Heinrich-Heine-Universität Düsseldorf

Institut für Bio- und Geowissenschaften IBG-1: Biotechnologie

Forschungszentrum Jülich GmbH

D-52426 Jülich, Germany

Phone: +49-2461-613716

Fax: +49-2461-612490

Email: karl-erich.jaeger@fz-juelich.de

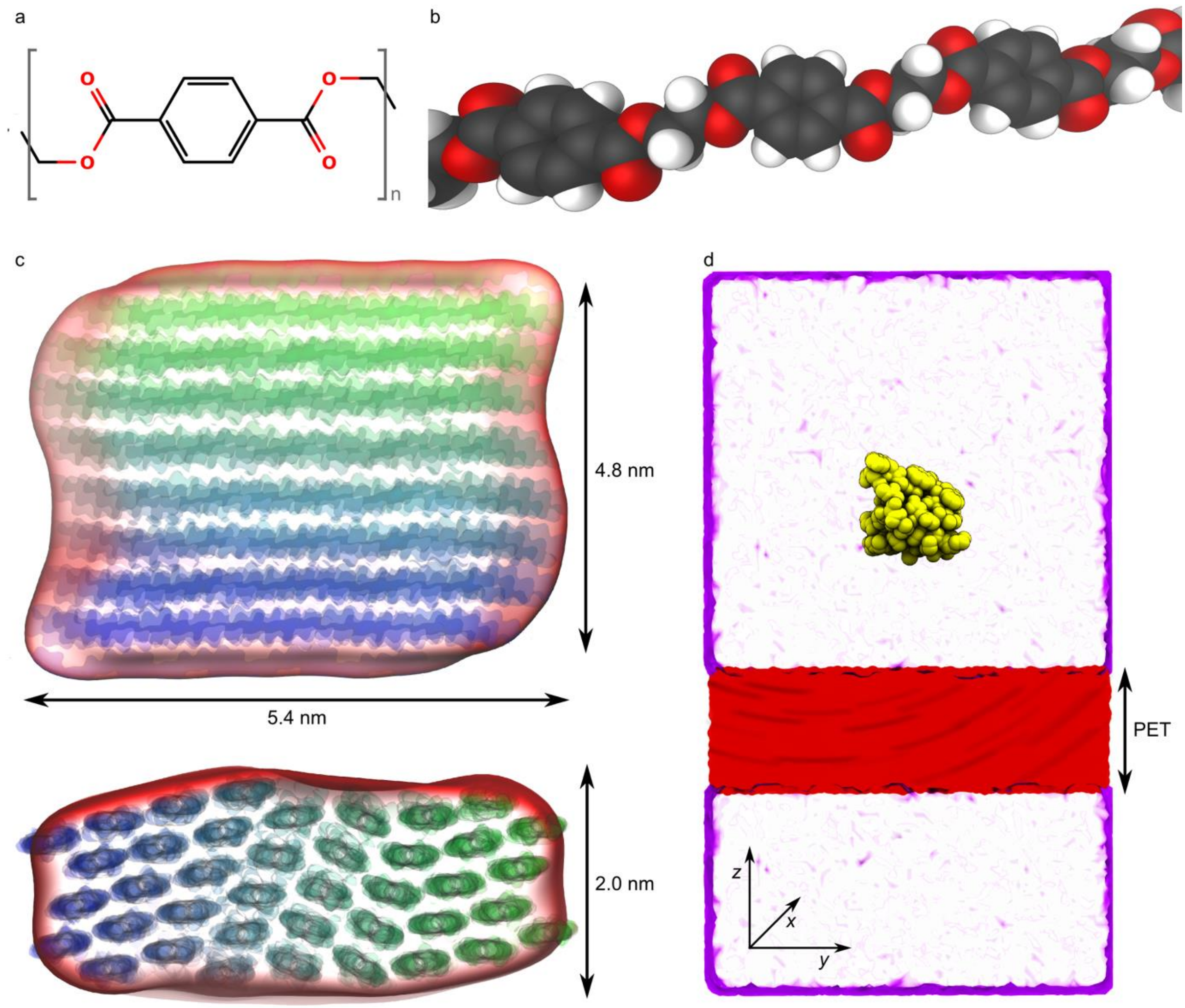


**Fig. S1** Poly(ethylene terephthalate) structure and PET modelling. (a) Chemical structure of a PET monomer. (b) A PET polymer chain contains alternating hydrophobic (phenyl) and polar (ester) groups. (c) The PET surface obtained using molecular dynamics was used to study peptide binding. (d) 2D projection of a typical simulation box consisting of an infinite PET surface (in x- an y-directions), surrounded by water in z-direction. Peptides were positioned in the water phase, no less than 1.0 nm from the PET surface.

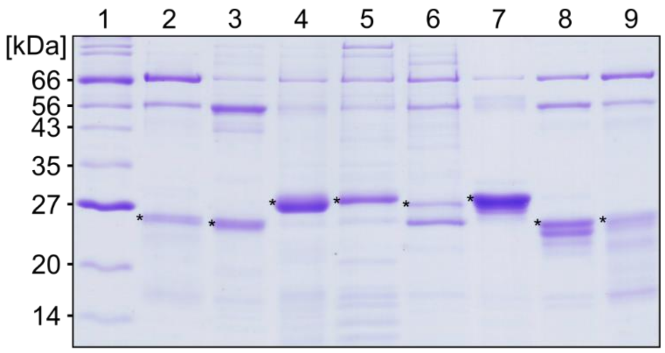


**Fig. S2** Analysis of purified peptide samples by SDS-polyacrylamide gel electrophoresis and subsequent staining with Coomassie Brilliant Blue. Lane 1: standard (Unstained Protein Marker, Broad Range; New England Biolabs), molecular masses are given on the left. Lane 2: *Tr*CBM1-Bs2-StrepII (21.2 kDa). Lane 3: *Pp*CBM1-Bs2-StrepII (21.1 kDa). Lane 4: *Ba*CBM2-Bs2-StrepII (25.9 kDa). Lane 5: *Bs*CBM2-Bs2-StrepII (26.6 kDa). Lane 6: *Ba*CBM5-Bs2-StrepII (23.1 kDa). Lane 7: *Bl*CBM5-Bs2-StrepII (23.8 kDa). Lane 8: ucCBM10-Bs2-StrepII (21.8 kDa). Lane 9: *Pa*CBM10-Bs2-StrepII (20.8 kDa). Full length protein is indicated by an asterisk. Lanes 2-9: 2 µg protein applied.

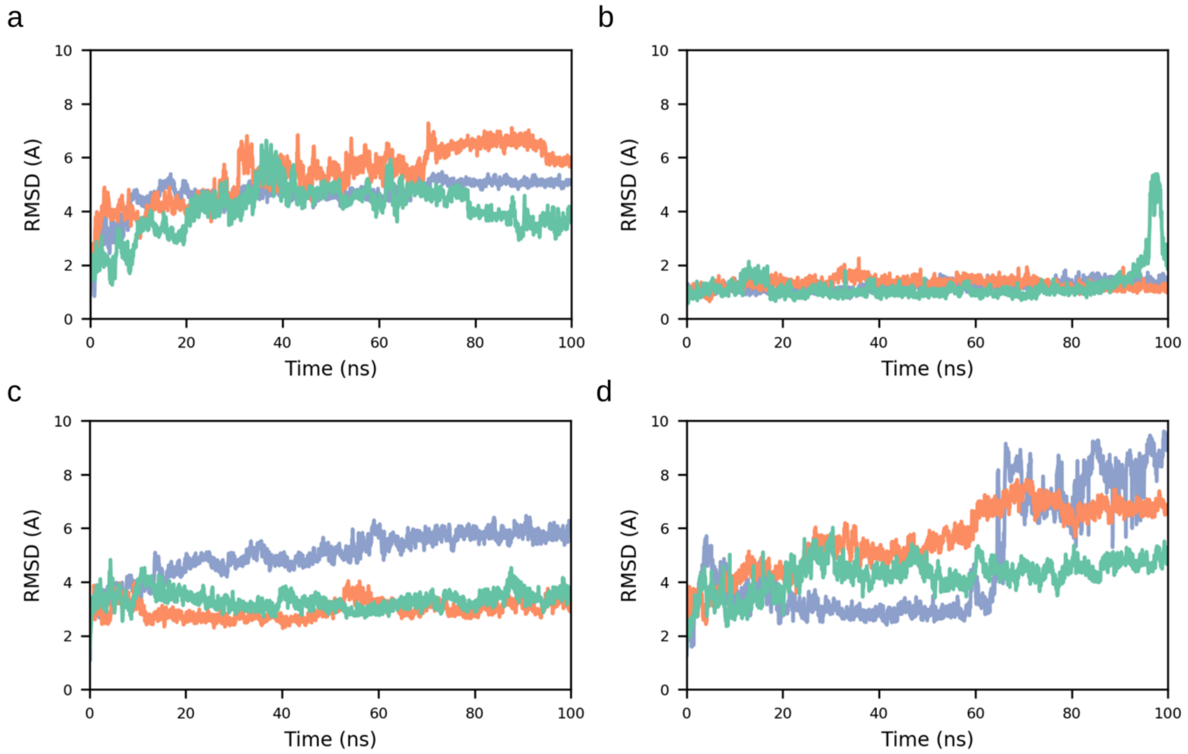


**Fig. S3** Peptide heavy atom backbone RMSD (aligned to the backbone atoms) from peptide–PET simulations with (a) *Tr*CBM1, (b) *Ba*CBM5, (c) *Ba*CBM2, and (d) W9A/W44A/W63A *Ba*CBM2 mutant. Three independent simulations are shown in blue, orange, and green, respectively.

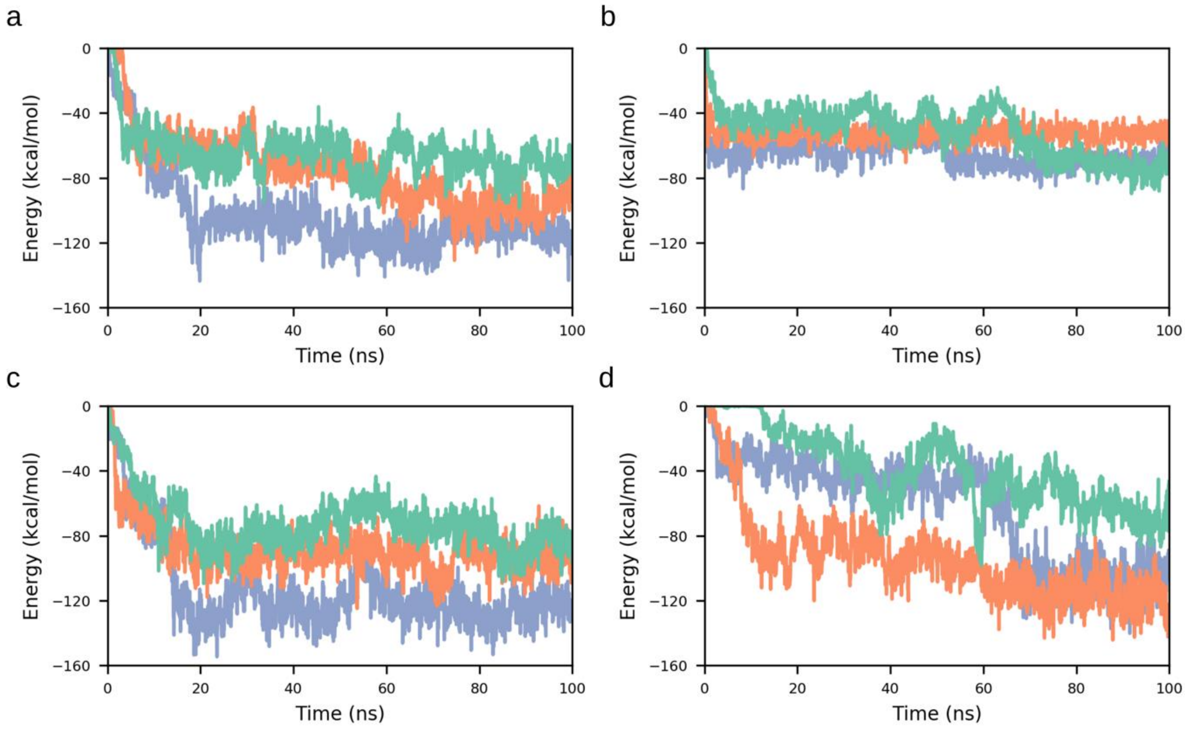


**Fig. S4** Peptide–PET nonbonded interaction energy (a) *Tr*CBM1, (b) *Ba*CBM5, (c) *Ba*CBM2, and (d) W9A/W44A/W63A *Ba*CBM2 mutant. Three independent simulations are shown in blue, orange, and green, respectively.

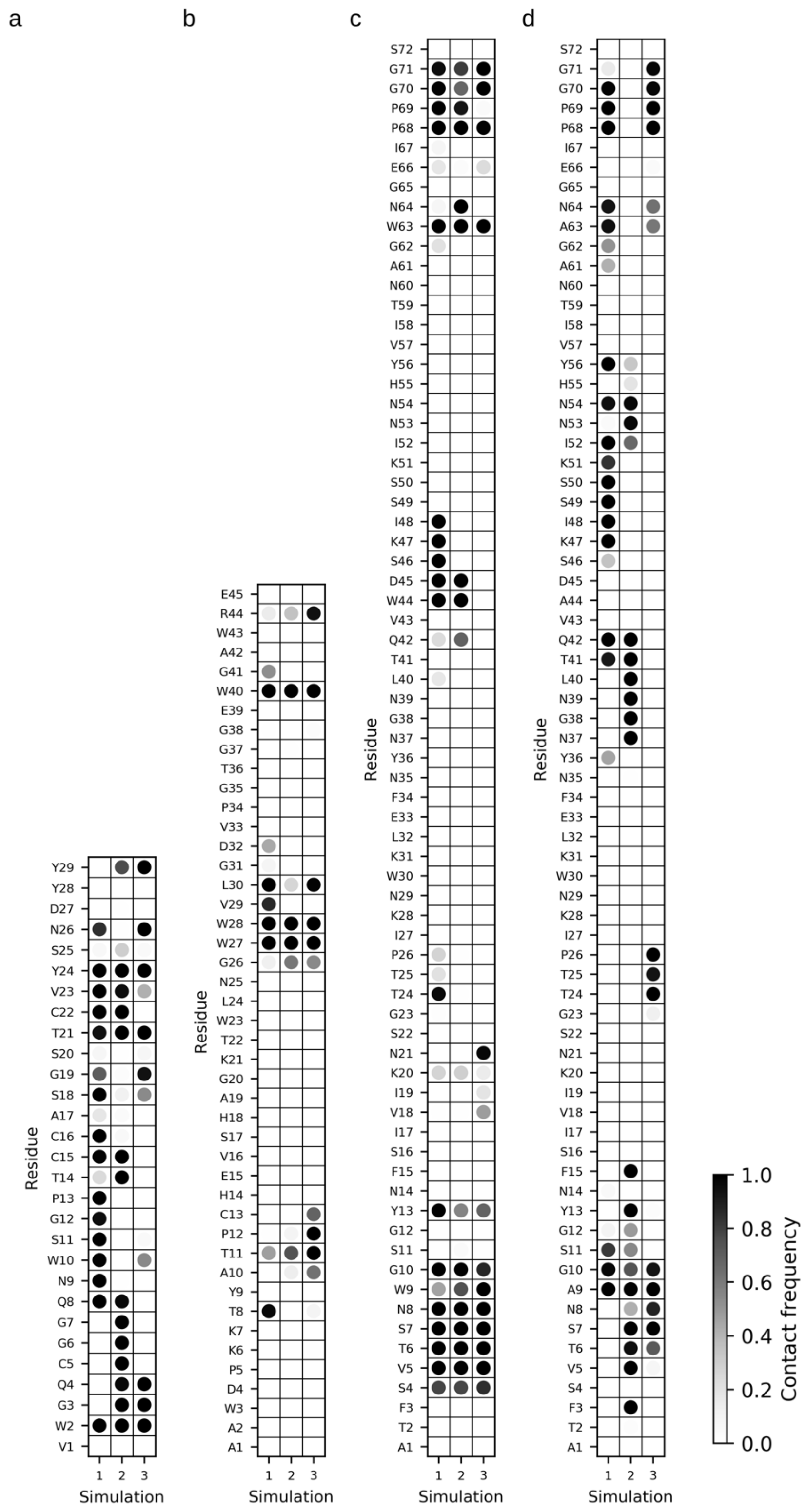

**Fig. S5** Peptide–PET contacts where PET is within 4 Å of (a) *Tr*CBM1, (b) *Ba*CBM5, (c) *Ba*CBM2, and (d) W9A/W44A/W63A *Ba*CBM2 mutant in three independent simulations. The colorbar represents the contact frequency during the last 10 ns of each MD trajectory.

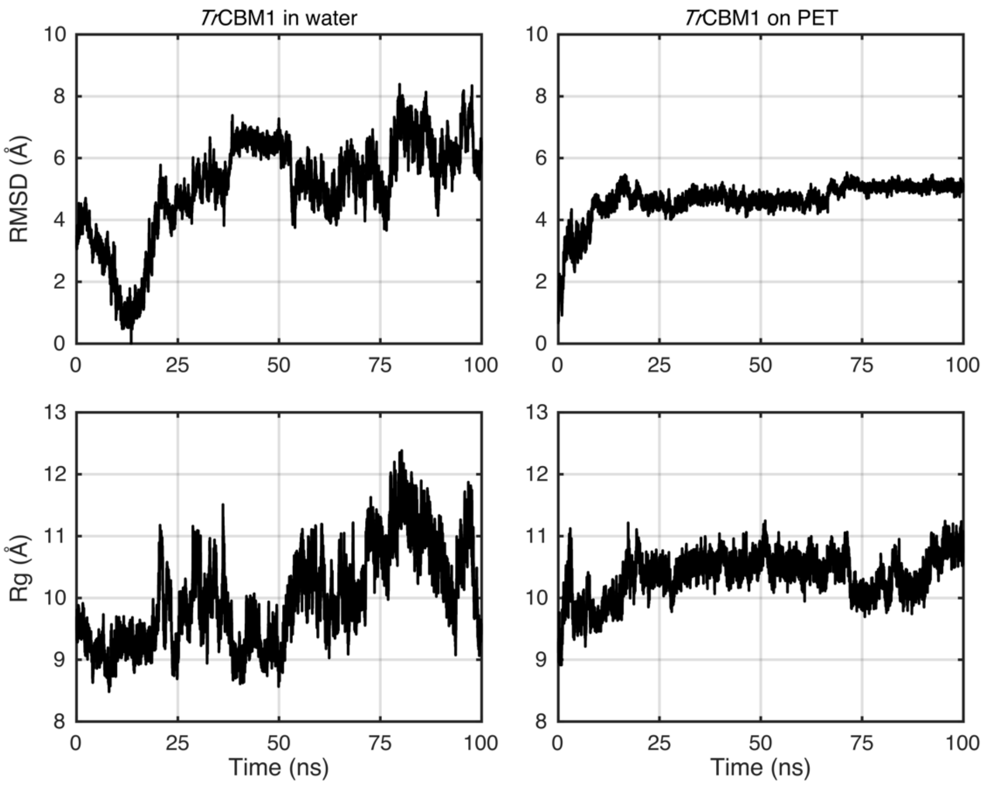


**Fig. S6** Comparison of the *Tr*CBM1 flexibility in bulk water and at the PET surface. Only one of three peptide–PET MD trajectories was shown.

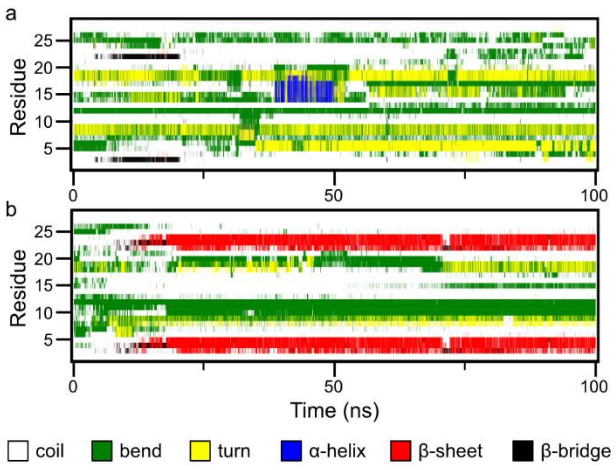


**Fig. S7** Development of the secondary structure elements in *Tr*CBM1 in the simulations (a) in bulk water and (b) at the PET interface. Only one of three peptide–PET MD trajectories was shown.

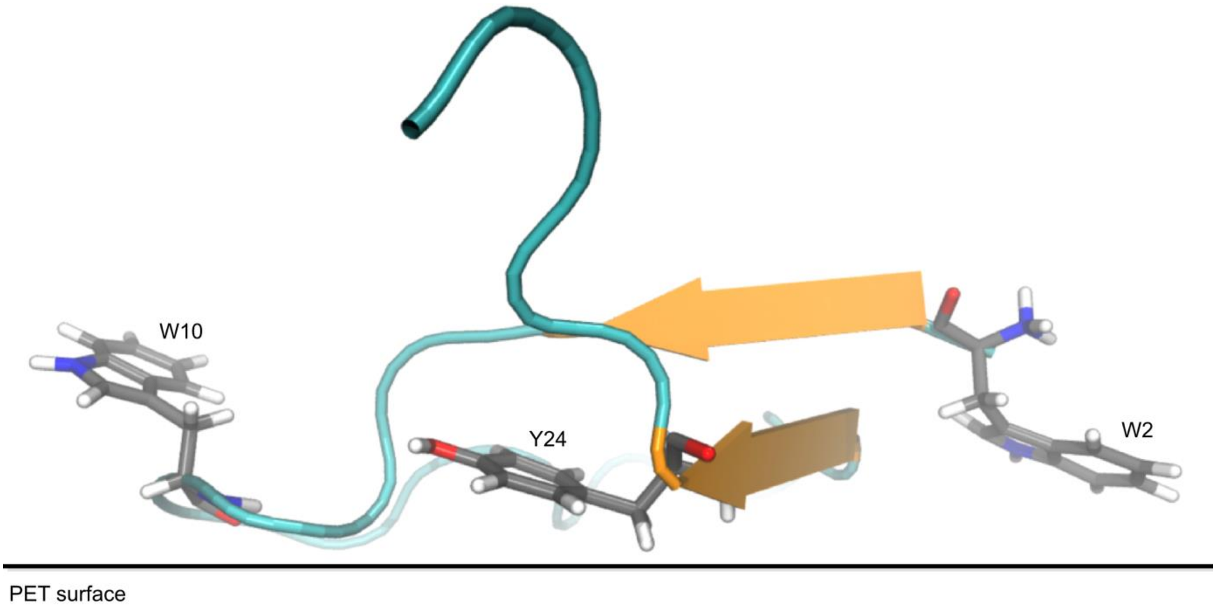


**Fig. S8** *Tr*CBM1 folds to a β-sheet at the interface with PET.
